# Development of a High-Density SNP-Based Linkage Map and Detection of QTL for β-Glucans, Protein Content, Grain Yield per Spike and Heading Time in Durum Wheat

**DOI:** 10.3390/ijms18061329

**Published:** 2017-06-21

**Authors:** Ilaria Marcotuli, Agata Gadaleta, Giacomo Mangini, Antonio Massimo Signorile, Silvana Addolorata Zacheo, Antonio Blanco, Rosanna Simeone, Pasqualina Colasuonno

**Affiliations:** 1 Department of Agricultural and Environmental Science, University of Bari “Aldo Moro”, Via G. Amendola 165/A, 70126 Bari, Italy; i.marcotuli@gmail.com (I.M.); pattybiotec@yahoo.it (P.C.); 2 Department of Soil, Plant and Food Sciences, Section of Genetic and Plant Breeding, University of Bari “Aldo Moro”, Via G. Amendola 165/A, 70126 Bari, Italy; giacomo.mangini@uniba.it (G.M.); massimoantonio.signorile@uniba.it (A.M.S.); silvanaaddolorata.zacheo@uniba.it (S.A.Z.); blanco17145@gmail.com (A.B.); rosanna.simeone@uniba.it (R.S.)

**Keywords:** durum wheat, single nucleotide polymorphism (SNP) mapping, QTL, β-glucans, grain protein content, grain yield per spike, heading time

## Abstract

High-density genetic linkage maps of crop species are particularly useful in detecting qualitative and quantitative trait loci for important agronomic traits and in improving the power of classical approaches to identify candidate genes. The aim of this study was to develop a high-density genetic linkage map in a durum wheat recombinant inbred lines population (RIL) derived from two elite wheat cultivars and to identify, characterize and correlate Quantitative Trait Loci (QTL) for β-glucan, protein content, grain yield per spike and heading time. A dense map constructed by genotyping the RIL population with the wheat 90K iSelect array included 5444 single nucleotide polymorphism (SNP) markers distributed in 36 linkage groups. Data for β-glucan and protein content, grain yield per spike and heading time were obtained from replicated trials conducted at two locations in southern Italy. A total of 19 QTL were detected in different chromosome regions. In particular, three QTL for β-glucan content were detected on chromosomes 2A and 2B (two loci); eight QTL controlling grain protein content were detected on chromosomes 1B, 2B, 3B (two loci), 4A, 5A, 7A and 7B; seven QTL for grain yield per spike were identified on chromosomes 1A, 2B, 3A (two loci), 3B (two loci) and 6B; and one marker-trait association was detected on chromosome 2A for heading time. The last was co-located with a β-glucan QTL, and the two QTL appeared to be negatively correlated. A genome scan for genomic regions controlling the traits and SNP annotated sequences identified five putative candidate genes involved in different biosynthesis pathways (*β-glucosidase*, *GLU1a*; *APETALA2*, *TaAP2*; *gigantea 3*, *TaGI3*; *14-3-3 protein*, *Ta14A*; and *photoperiod sensitivity*, *Ppd-A1*). This study provides additional information on QTL for important agronomic traits that could be useful for marker-assisted breeding to obtain new genotypes with commercial and nutritional relevance.

## 1. Introduction

Durum wheat (*Triticum turgidum* L. subsp. *Durum*) is the tenth most widely grown crop on the planet with production of more than 36 million metric tons. It is commonly cultivated on both south and north shores of the Mediterranean Sea, with the largest proportion of the global production focused there. This crop was domesticated at the origin of human civilization and Phoenicians traded it along these waters in historical times.

Durum is an excellent dietary source for its high levels of fibre, protein and carotenoids. It is also a significant source of rare minerals like magnesium and selenium. The grain quality of wheat varieties is of paramount importance in determining the nutritional value of the derived end products. The price of durum grain is linked to consumer appreciation and overall dietary quality; better quality is linked with higher prices. Considerable variation exists in grain quality of different varieties and this difference is often due to heritable genetic components, even though the environment and management practices (especially genotype × environment and genotype × agronomic management interactions) also have significant effects [1]. Many studies aimed at improving wheat quality have focused on the grain/flour value of breeding lines, environmental effects, and end-use processing quality [2]. Thus, there are different ways of considering the general words “wheat quality”. The commercial wheat quality value is primarily determined by the milling rate, the quantity of wheat flour derived from 100 kg of seeds. The maximum quantity of flour obtained is affected by seed size, thousand kernel weight, ash mass (or mineral content) and percentage of defective seeds (such as sprouted, small or white seeds, and darkness of grain caused by black point). However, at the technological level, wheat quality is determined by the strength of the flour protein when mixed with water to make dough, and the quantity of water required to produce workable dough. The protein quantity and quality influence the characteristics of the final food products [3]. For example, high protein content brings high water absorbance and increases the shelf life of the products. Another important technological parameter is fibre content, which is linked with many health benefits including those associated with immunomodulatory activity, blood cholesterol, faecal bulking effects, mineral absorption, prebiotic effects, and reduced type II diabetes [4]. The principal components of fibre are β-glucans and arabinoxylans. Even though β-glucans are a minor component of wheat cell walls they are among the most important portion of the fibre-soluble fraction with beneficial effects on human health. Furthermore, the hydrocolloid properties of β-glucan may positively affect bread quality by extending its shelf life in contributing to sustained firmness and prevention of dehydration during storage [5]. Mohamed et al. [6] reported a delay in the hardening of bread containing β-glucan during storage, whereas Kurek et al. [7] found a positive effect of β-glucan on product quality due to increased water retention in the bread. Moreover, they have potential use as functional ingredients to improve mouth-feel of low-fat dairy products [4]. β-glucan and grain protein contents, as well as yield components, are controlled by complex genetic systems that are influenced by environmental factors and management practices. During recent decades, breeding programs have been more focused on quality traits, such as protein and fibre content. Nevertheless, maintenance of yield remains a major objective.

Due to the importance of wheat as a major crop, its genome has been extensively studied. A high-density wheat single nucleotide polymorphism (SNP) iSelect array recently developed by Wang et al. [8] comprises approximately 90,000 gene-associated SNPs and provides a dense coverage of the wheat genome. Many wheat germplasm collections [9,10,11,12] and biparental cross populations [2,13] have also been used to achieve SNP-based maps and SNP consensus maps in both bread wheat and durum [7,14,15], which were also used in quantitative trait studies.

Many studies have reported Quantitative Trait Loci (QTL) for protein content [16], β-glucan content [10] and yield [2], and correlations between protein and yield components in durum wheat, largely investigated in segregating populations, germplasm collections and recurrent selection material [3,17]. Unfortunately, there is a lack of information concerning β-glucan content in relation to protein content and yield components. Therefore, our objectives were to: (a) evaluate the genetic variation in β-glucan and protein content, grain yield per spike and heading time in a recombinant inbred line (RIL) population derived from a cross between two elite durum wheat cultivars; (b) develop a high-density SNP-based genetic linkage map; (c) identify and characterise QTL for β-glucans and protein content, grain yield per spike and heading time; and (d) identify candidate genes associated with the analysed traits.

## 2. Results

### 2.1. Field Trait Analysis

**Figure 1:**
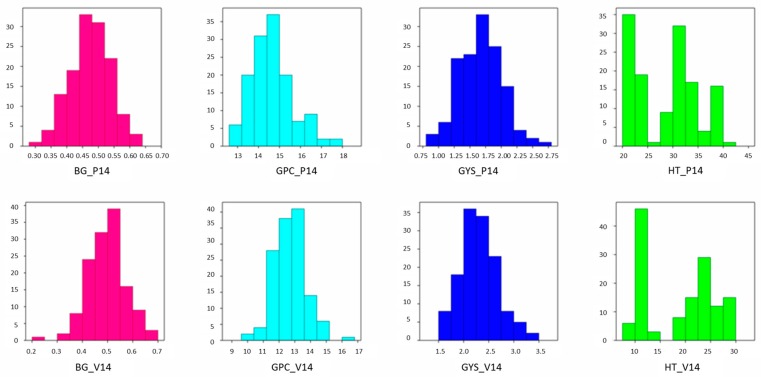
Frequency distribution of β-glucan content (BG), grain protein content (GPC), grain yield per spike (GYS) and heading time (HT) in the Duilio × Avonlea recombinant inbred lines (RIL) population grown at Policoro and Valenzano in 2014. Reported values are means of three biological replicates.

Genetic variation in β-glucan content (BG), grain protein content (GPC), grain yield per spike (GYS) and heading time (HT) was assessed in a Duilio × Avonlea (D × A) RIL population grown at Policoro (Matera, Italy) and Valenzano (Bari, Italy) in 2014 (Figure 1).

All traits were determined in three field replicates in order to evaluate the environmental influence. Descriptive statistics (general mean for each parent and RIL, range of the RIL population, genetic variance, and broad-sense heritability) are reported in Table 1.

The phenotypic data for yield and adaptive components highlighted considerable differences between the parents and significant genetic variation in the RIL population in both environments. Avonlea was later heading and had lower GYS than Duilio. Qualitative trait data showed differences in BG, with cv. Avonlea having higher content, whereas GPC was similar for both parents. For all traits, the range of the RIL population was much larger than the range of the parental lines, suggesting transgressive segregation and that favourable alleles were present in both parents.

The means of the RIL population for BG were near Duilio, but with a big range of variability (0.30–0.63% and 0.22–0.68%) in both the environments with a typical pattern of a quantitative trait. The broad-sense heritability estimates (genotype mean basis) ranged from 0.80 to 0.82, indicating the stability of the trait and the phenotypic expression mainly due to genotypic effects.

Protein content in the RIL population ranged from 10.2 to 17.6% with mean values corresponding to the parental values. Broad-sense heritability (0.52 and 0.44) indicated the degree to which phenotypic expression was influenced by genetic versus environmental effects.

Similarly low estimates of heritability were observed for GYS. Moreover, GYS data showed a wide range of variability in the D × A RIL population (0.94–2.64 g and 1.58–3.47 g), typical of a quantitative trait and with mean values corresponding to the parental means. There was wide variation and transgressive segregation for HT with a range of values from 9 to 41. The heritability was 0.99–0.98, indicative of a high degree of stability for this trait. Contrary to the other traits analysed, heading date was bimodally distributed.

Correlation analyses (Table 2) between the various traits indicated significant and positive relationships for GPC and HT in Valenzano 2014 (*p* ≤ 0.01). A significant and negative correlation was detected between GPC and BG (*p* ≤ 0.01) in Valenzano 2014. Negative correlations between GPC and GYS were observed at both locations (*p* ≤ 0.001).

### 2.2. A Duilio × Avonlea Linkage Map

The D × A RIL population was genotyped with the wheat 90K SNP iSelect assay [7]. Of 81,587 assays, 5577 (6.8%) failed and 69,692 (85.4%) were monomorphic across the mapping population. The remaining 6831 (8.37%) were polymorphic; however, 513 had more than 10% missing data and were excluded from further analyses. Hence, 6318 markers were used for map construction.

**Figure 2:**
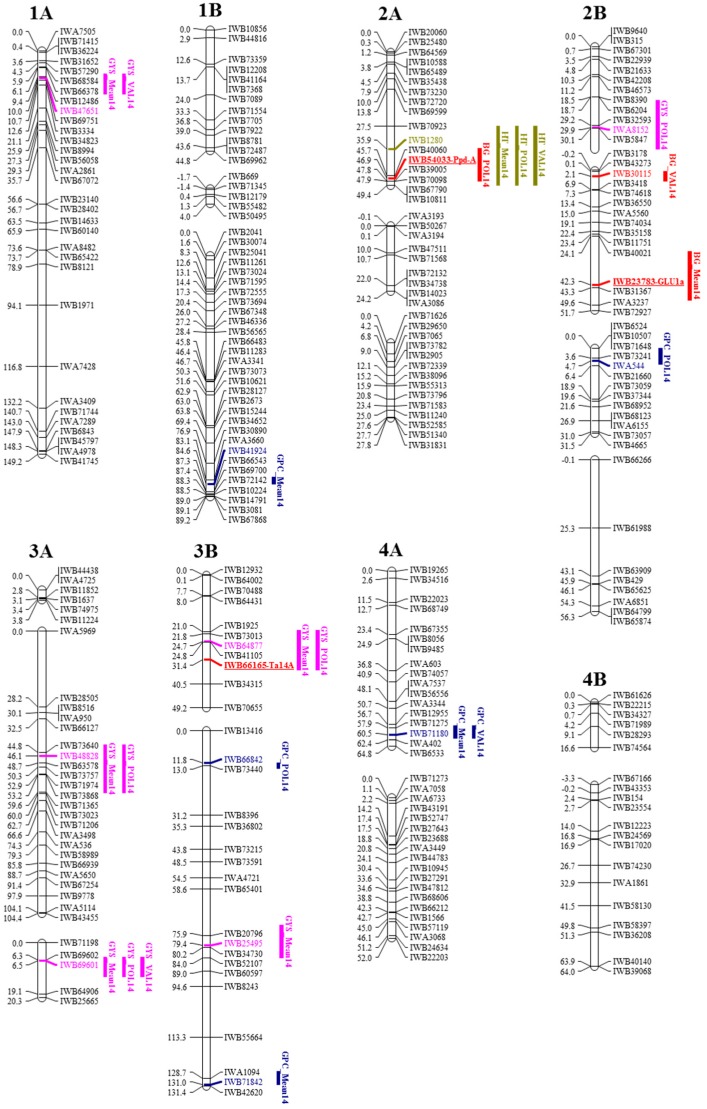

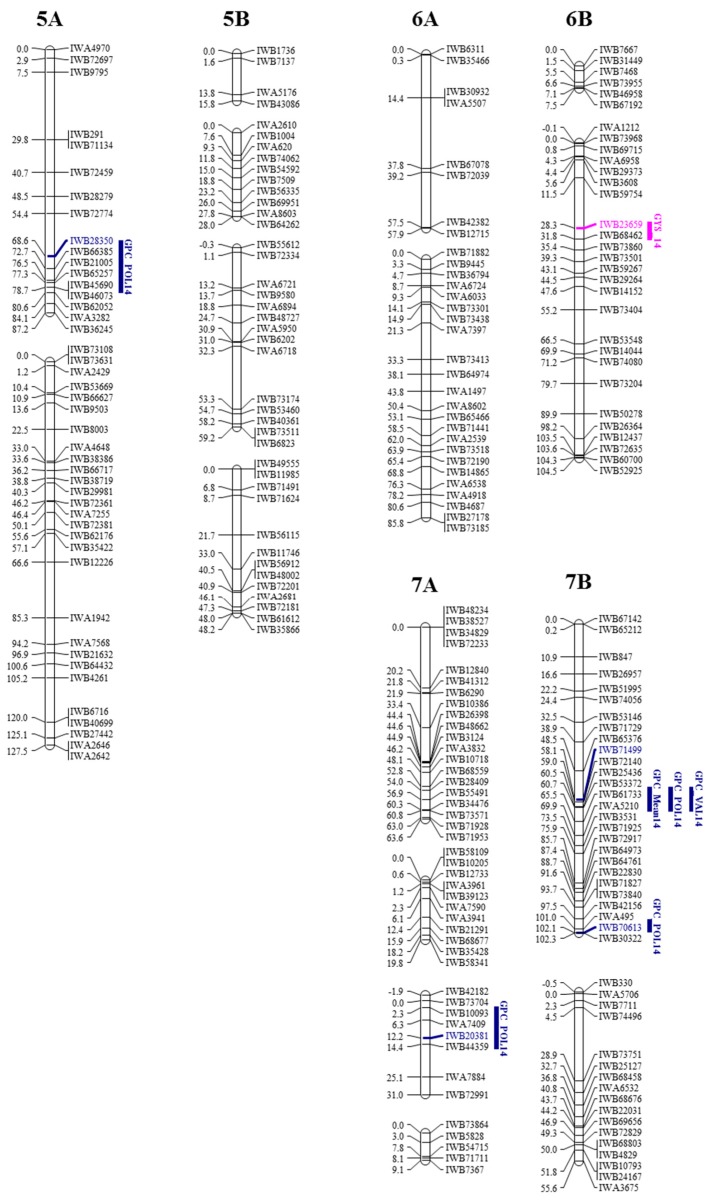
Schematic representation of the durum wheat chromosomes and Quantitative Trait Loci (QTL) for BG, GPC, GYS and HT detected in 134 RILs derived from Duilio × Avonlea. Map positions are given in cM (on the left). Each chromosome is represented by single nucleotide polymorphism (SNP) markers (on the right) mapped on durum wheat consensus maps [13,15]. QTL are represented by bars on the right of each chromosome bar. QTL names indicate the trait (BG, GPC, GYS and HT) and the environment in which the QTL was detected (Mean14, mean in 2014; POL14, Policoro in 2014; VAL14, Valenzano in 2014); the closest SNP marker is indicated in colour and the candidate gene is reported next to the corresponding marker. Solid bars represent QTL declared at LOD score ≥ 3.0.

Of 6318 loci, 5444 (6.7% from the original SNP dataset) assembled in 36 linkage groups when using a LOD score ≥ 6, and 874 remained unlinked or assembled in small linkage groups (LGs) without a chromosomal anchor locus. Linkage groups were assigned to the A and B genome chromosomes using the physical position of SNP reported in Colasuonno et al. [13] and the SNP anchor loci reported in the durum consensus map of Maccaferri et al. [15] (Figure 2, Table S1).

A total of 2904 (53.3%) markers were localised on the B genome with a total length of 1001.3 cM, whereas 2540 (46.6%) were mapped on the A genome (total length 961.2 cM). The entire map covered 1962.5 cM with an average chromosome length of 140.2 cM. The lengths of individual chromosomes varied from 80.6 cM (chromosome 4B) to 214.7 cM (chromosome 5A). Chromosomes 2A and 7A showed the highest number of markers per cM (4.2 and 4.6, respectively), and chromosome 3B had the lowest marker density (1.6) (Table 3).

Linkage analysis showed a high number of co-segregating SNP (1596). The SNP markers were generally well distributed throughout the genome, although some chromosomes exhibited higher densities.

The overall SNP density was 2.7 markers/cM, with a maximum of 4.6 for chromosome 7B and a minimum of 1.6 for chromosome 3B. The recombinant frequency was 49.7%. The percentage of mapped SNPs showing segregation distortion at *p* ≥ 0.05 was 5.6%.

### 2.3. Detection of QTL for β-Glucan Contents, Protein Content, Grain Yield per Spike and Heading Time

Identification of QTL associated with BG, GPC, GYS and HT was carried out based on the genetic map obtained as previously described. Composite interval mapping (CIM) as proposed by Zeng [18] identified three QTL for β-glucans with LOD values ≥3.0 on chromosomes 2A and 2B, consistent in the mean of the two environments and in one location (Policoro), respectively (Figure 2, Table 4). For additive QTL effects, positive and negative signs indicate the relative trait contributions from Avonlea and Duilio, respectively. The QTL on chromosome 2A had a negative effect due to the Duilio alleles, whereas both QTL on chromosome 2B showed positive effects by Avonlea alleles.

Eight QTL for GPC were located on chromosomes 1B, 2B, 3B (two loci), 4A, 5A, 7A and 7B. Positive alleles in Avonlea were on chromosomes 1B, 3B (both loci) and 7B, and those on 2B, 4A and 5A were from Duilio. The 7B QTL (*QGpc.mgb-7B.1*) was detected in both two environments and the mean.

QTL for GYS were detected on chromosomes 1A, 2B, 3A (two loci), 3B (two loci) and 6B with *QGys.mgb-1A.1*, *QGys.mgb-3A.1* and *QGys.mgb-3B.1* having positive effects from Avonlea and a negative effect for the rest of the loci.

Regression analysis allowed identification of a single region on chromosome 2A associated with HT that was consistent in both environments with a high LOD score of 25.5.

### 2.4. Candidate Genes Related to All the Detected QTL

Sequences of SNP markers in Wang et al. [7] were used to identify potential candidate genes associated with QTL for BG, GPC, GYS and HT detected in the D × A population. All the sequences of the SNP markers located in the QTL regions were analysed for annotated genes or proteins involved in each considered pathway in wheat. For BG, the marker *IWB23783* located on chromosome 2B corresponded to β-glucosidase (*GLU1a*) (Table 5).

For GYS, candidate genes were detected on chromosomes 2B, 3A and 3B. Since the low number of SNPs in the 2B QTL region limited this study, we considered the same region in the consensus durum map [15]. This allowed us to detect the *APETALA2* (*TaAP2*) gene corresponding to the *wmc213-2B* and *wmc243-2B* markers. Both SSRs mapped at 4.0 cM from the closest associated (*IWA8152*) marker. The *gigantea 3* (*TaGI3*) gene showed sequence similarity with the *IWB65703* marker (contig_3AS_200_3324175) at 0.6 cM from *IWB48828* in the 3A QTL region. The 14-3-3 protein (*Ta14A*) gene, identified by the SNP marker *IWB66165*, mapped in the 3B QTL region at 7.0 cM from the closest SNP marker *IWB64877* (Table 5).

The heading time gene *QHt.mgb-2A.1* appeared to be associated with the photoperiod sensitivity (*Ppd-A*) gene through SNP marker *IWB54033* in the chromosome 2A QTL region.

## 3. Discussion

The recent emphasis on grain quality over grain quantity has shifted the major objective of wheat breeding programs to increasing nutrient properties while maintaining or increasing grain yield in lines to be released for commercial production. For a long time the major quality objective in durum breeding has been increased GPC [19] and yellow pigment content [9], but recently, more attention has been given to fibre [11], for which the main objective has been improved BG [10]. Different components contribute to grain yield, and among them the fundamental one is grain yield per spike, which is generally negatively correlated to GPC [2]. Another trait correlated with yield is HT, which in cereal crops is determined by vernalisation requirement, photoperiod sensitivity, and narrow-sense earliness or earliness per se [20]. Thanks to the recently released consensus linkage maps for bread wheat [8] and durum [15], development and saturation of genetic maps for detection of QTL underlying agronomic traits has rapidly increased in genetics studies.

In the present work, we evaluated BG, GPC, GYS and HT in an Italian cv. Duilio × Canadian cv. Avonlea RIL population grown in two field trials and developed a high-density linkage map. The total map length was 1962.5 cM, and included 5444 SNP markers, of which 374 SNPs were used to anchor the linkage groups (LGs) to chromosomes. A good coverage and representation of all 14 durum chromosomes was obtained, but the presence of gaps (over 20 cM) led to 36 LGs. Despite the high density of polymorphic markers detected using the 90K SNP assay, linkage groups ranging from 20 to 33 cM have been reported in previous SNP maps [13,21,22,23]. This fragmentation observed for the D × A map could be dependent upon the strict conditions used in determining the marker order (LOD score > 6 and SNP markers with less than 10% missing data). Despite the numerous LGs, our map represented a useful basis for studies on marker-assisted selection and positional cloning. The D × A map had a higher length (1962.5 cM) than previous maps [2,3,13], but a lower marker density (2.7 cM). The average number of mapped markers per chromosome was 389, with a range from 230 SNPs on chromosome 4B to 536 on chromosome 7B. More markers were mapped on the B genome as observed in other linkage studies [2,3,13]. Chromosome 1A was represented by only one LG [15].

A total of 19 QTL for the examined traits were detected, including three for BG, eight for GPC, seven for GYS, and one for HT.

BG QTL were mapped on chromosomes 2A and 2B, as previously reported by Marcotuli et al. [10], but in different chromosome positions. *QGbg.mgb-2A.1* was co-located with *QHt.mgb-2A.1* controlling HT and with a strong and negative effect on the trait (−9.96) due to Duilio contributing the negative effect. Previous work on yield components reported a major QTL for HT on chromosomes 2BL with negative alleles on the trait [24,25]. We found no significant correlation between BG and HT. Similar results were reported in oat [26,27] and barley [28,29]. Due to the paucity of studies on BG in wheat at any ploidy level, the present data provide some knowledge and understanding of the trait in durum wheat.

A large number of studies have identified major and minor QTL for GPC in wheat [16,30,31]. We mapped eight QTL on chromosomes 1B, 2B, 3B, 4A, 5A, 7A and 7B in the present RIL population. The major stable QTL on chromosome 7B was repeatedly detected in common wheat and durum [16,31,32]. The other QTL on chromosomes 1B [24,33], 3B [30,33,34], 4A [30,31] and 7A [30] were not stable across environments, but were already reported in the literature.

QTL for GYS have been reported on almost all wheat chromosomes [1,33,35,36,37,38,39,40,41,42,43,44]. Most of these studies identified several grain yield QTL; however, the majority were detected in only single environments, and QTL detected in more than one environment showed considerable variation in magnitude of effect [1,36,37,41,42,43]. Our study detected QTL on chromosomes 1A, 2B, 3A, 3B and 6B as already reported in literature [2,45,46,47], of which *QGys.mbg.3A.1* was consistent in both environments.

To identify coding sequences in regions associated with QTL, BLAST analyses on the NCBI database [48] were carried out. No gene co-located with the BG QTL *QGbg.mgb-2A.1* and *QGbg.mgb-2B.1*, whereas the mapped SNP *IWB23783* associated with *QGbg.mgb-2B.2* corresponded to β-glucosidase 1a (*GLU1a*), a member of the glycoside hydrolase family 1 (GH1). This enzyme catalyses hydrolysis of the glycosidic linkage in glycosides, leading to formation of a hemiacetal or hemiketal sugar and a corresponding free aglycon [49]. We also detected the *photoperiod sensitivity* (*Ppd-A1*) gene in the QTL *QHt.mgb-2A.1* region. The contig sequence of *IWB54033* located in the 2A QTL corresponded to the *Ppd-1A* sequence, confirming previous results on the relationship between photoperiod response and HT [50,51]. No marker located in gene sequences for GPC was identified, but previous studies reported genes involved in the nitrogen pathway located in the same position of QTL reported in the present work [2,52,53,54,55].

Bioinformatics analysis of markers located in QTL region *QGys.mgb-2B.1* for GYS detected no candidate gene associated with the trait. Because previous studies reported a gene (*APETALA2*) associated with a QTL for grain yield in different species [56,57,58,59], we decided to use all the markers (SNPs and Simple Sequence Repeat, SSR) from the durum wheat consensus map [15] co-locating with all the SNP mapped in the 2B chromosome interval 19.9–37.9 cM of the D × A map. Due to the higher number of considered markers, we identified the *APETALA2* (*TaAP2*) gene associated with the SNP marker *IWA8152*.

In *Arabidopsis*, Aukerman and Sakai [56] and Jung et al. [57] demonstrated that overexpression or knockout of the *APETALA 2-like* (*AP2*) gene delayed flowering time or caused earlier flowering, respectively. Similar studies were conducted in rice [58], barley [59] and wheat [59]. Another gene involved in flowering promotion and associated with *QGys.mgb-3A.2* was *gigantea 3* (*TaGI3*) [60]. A third gene detected in the region controlling GYS was *14-3-3 protein* (*Ta14A*) associated with two SNPs (*IWB3714* and *IWB11529*). *Ta14A* was strongly expressed in leaf and stem tissues, but was undetectable in roots, suggesting tissue-specific functions [61].

## 4. Materials and Methods

### 4.1. Genetic Materials and Field Experiments

A set of 134 F_2:7_ RILs were developed from a cross between Italian cultivar Duilio and Canadian cultivar Avonlea. After the last generation of selfing, each line was harvested in bulk to provide seeds for replicated trials and DNA extraction. The parental lines and RILs were evaluated for β-glucan (BG), grain protein content (GPC), grain yield per spike (GYS), and heading time (HT) in replicated trials at Policoro (metropolitan city of Matera) and Valenzano (metropolitan city of Bari) in southern Italy in 2014.

A randomised complete block design with three replications was used in field experiments. Plots consisted of 1 m rows spaced 30 cm apart; each plot had 80 germinated seedlings. During the growing season, 10 g of nitrogen per m^2^ were applied at flowering stage and standard cultivation practices were adopted.

Plots were hand-harvested at maturity and GYS was determined by dividing grain yield by the number of spikes (about 70–80 spikes) per row.

β-glucan (percentage *w*/*w*) content in the whole grain was assayed using a Mixed-Linkage β-Glucan Assay Kit (Megazyme International Ireland Ltd, Wicklow, Ireland) based on the method by McCleary and Codd [62] and included the industrial standard for barley (4.1% β-glucan). GPC, expressed as a percentage of protein on a dry weight basis, was determined on a 2 g sample of whole-meal flour using an InfraAlyzer spectrophotometer (Technicon Industrial Systems, Tarrytown, NY, USA) (that is, using near-infrared reflectance spectroscopy).

HT was recorded as the number of days from 1 April 2014, to 50% ear-emergence, corresponding to stage 55 on the Zadoks et al., scale [63].

### 4.2. SNP Genotyping

Fifty ng/µL of genomic DNA from each RIL and parental line (Duilio and Avonlea) was analysed with the wheat 90K iSelect array [8]. Genotyping was performed at TraitGenetics GmbH (http://www.traitgenetics.de [64]) following the manufacturer’s recommendations as described in Akhunov et al. [65]. The genotyping assays were carried out using the Illumina iScan reader and performed using GenomeStudio software version 2011.1 (Illumina, San Diego, CA, USA).

### 4.3. Segregation Analysis and Map Construction

Chi-squared tests were used to determine the goodness-of-fit at *p* > 0.001 of segregation ratios to expected 1:1 ratios for each SNP. All markers with more than 10% missing data, or segregating presence/absence in the mapping population were excluded from further analysis. Linkage analysis between markers and determination of the linear order of loci was performed by JoinMap 4.0 (Kyazma, Wageningen, Netherlands) [66] using the regression mapping algorithm. Grouping was performed using the independence LOD parameter, with groups presenting a LOD 6. The Haldane mapping function was used to calculate map distances [67]. Physically mapped SNP markers [13] and SNP data from the durum consensus maps [15] were used as anchor loci and for assigning linkage groups to specific chromosomes. Linkage groups were named according to the wheat chromosome nomenclature followed by a number linkage groups within chromosomes.

### 4.4. Statistical Analysis, and QTL and Genes Detections

Analysis of variance was carried out for each trait using GenStat (version 18, VSN International Ltd, Hemel Hempstead, UK,). QTL detection was performed in QGene 8.3.16 using composite interval mapping [18]. A marker-trait association was considered significant when one or more markers was associated with a trait at a threshold of −log_10_(*p*) ≥ 3.0, determined by modified Bonferroni correction. For additive effects, positive and negative signs for estimates indicated the contributions of Duilio and Avonlea, respectively, toward higher trait values. Regression analysis implemented in QGene was used for HT. Graphical representations of linkage groups and QTL were carried out using MapChart 2.2 software (SolarWinds, Austin, TX, USA) [68].

Putative candidate genes associated with QTL for BG, GPC, GYS and HT were identified using SNP sequences from Wang et al. [8] blasted against *Triticum* sequences annotated in NCBI [69] and contig sequences from the Unité de Recherche Génomique Info (URGI) website [70].

## 5. Conclusions

In the present study, we report a high-density SNP-based genetic map for the Duilio × Avonlea durum wheat RIL population. It comprised 5444 SNP loci. This high-resolution map and the data on β-glucan content, protein content, grain yield per spike, and heading time evaluated in two environments provided increased knowledge of BG in durum wheat and its interaction with other quantitative traits. The three QTL for BG were influenced by environmental differences. The QTL on chromosome 2B was coincident with the *β-glucosidase 1a* gene involved in the BG pathway. The QTL for BG on chromosome 2A was coincident with HT providing additional information on trait correlations. The marker-trait associations for GPC and GYS confirmed previous data reported in the literature on the polygenic nature of the traits and correspondent QTL in both common and durum wheat. The overall results provide a basis for marker-assisted selection for BG in breeding programs.

## Figures and Tables

**Table 1:** Descriptive statistics of BG, GYS, GPC and HT for the two parental lines (Avonlea and Duilio) and the RIL population D × A grown at Policoro (Matera) and Valenzano (Bari) in 2014. The genetic variance (s^2^_G_) and broad-sense heritability (h^2^) of the traits are reported for the RIL population.

Trait	Environment	
	Policoro 2014	Valenzano 2014
**β-glucan (%)**		
Duilio	0.48	0.48
Avonlea	0.56	0.59
RIL mean	0.47	0.49
Range	0.30–0.63	0.22–0.68
s^2^_G_	0.004	0.004
h^2^	0.80	0.82
**Grain protein content (%)**		
Duilio	14.3	12.0
Avonlea	13.9	12.1
RIL mean	14.63	12.7
Range	12.6–17.6	10.2–16.5
s^2^_G_	0.611	0.550
h^2^	0.52	0.44
**Grain yield per spike (g)**		
Duilio	1.71	2.37
Avonlea	1.93	2.55
RIL mean	1.67	2.31
Range	0.94–2.64	1.58–3.47
s^2^_G_	0.068	0.091
h^2^	0.47	0.53
**Heading time (days)**		
Duilio	20	12
Avonlea	38	26
RIL mean	29	19
Range	20–41	9–30
s^2^_G_	35.163	44.044
h^2^	0.99	0.98

**Table 2:** Correlations between BG, GPC, GYS and HT for the Duilio × Avonlea RIL population grown at Policoro and Valenzano in 2014.

Trait	Environment	GPC	GYS	HT
BG	Policoro	−0.04	0.12	−0.10
	Valenzano	−0.24 **	−0.06	−0.02
GPC	Policoro		−0.36 ***	−0.11
	Valenzano		−0.38 ***	0.20 **
GYS	Policoro			0.16
	Valenzano			0.05

** significant at *p* = 0.01; *** significant at *p* = 0.001.

**Table 3:** Number and distribution of SNP markers in the Duilio × Avonlea map.

Chromosome	Number of Linkage Groups	Number of SNPs	Chromosome Length (cM)	Number of SNPs/cM
1A	1	302	149.2	2.0
1B	3	522	137.9	3.8
2A	3	422	101.4	4.2
2B	4	492	169.6	2.9
3A	3	283	128.5	2.2
3B	2	294	180.6	1.6
4A	2	273	116.8	2.3
4B	3	230	80.6	2.8
5A	2	473	214.7	2.2
5B	4	454	151.2	3.0
6A	2	287	143.7	2.0
6B	2	376	123.5	3.0
7A	4	500	106.9	4.6
7B	2	536	157.9	3.4
Total genome A	17	2540	961.2	2.6
Total genome B	19	2904	1001.3	2.9
Total genomes AB	36	5444	1962.5	2.7

**Table 4:** Marker-trait associations for BG, GPC, GYS, and HT determined for each location (Policoro and Valenzano) and for the mean values of the two environments. QTL, closest marker, chromosome locations, interval (cM), marker effects (additive effect with positive and negative value indicating contribution of parent Avonlea and Duilio, respectively), LOD value (logarithm-of-odds) ≥3.0, and *R*^2^ (% of phenotypic variation explained by QTL) are reported for each SNP associated with each trait.

QTL	Closest Marker	Chrom	QTL Interval	Mean Across Environments			Policoro			Valenzano		
			cM	Effect	LOD	*R*^2^	Effect	LOD	*R*^2^	Effect	LOD	*R*^2^
**β-glucan content**												
*QGbg.mgb-2A.1*	IWB1280	2A	(35.8–48.0)	−0.03	4.5	0.14	−0.02	6.1	0.19	-	-	-
*QGbg.mgb-2B.1*	IWB30115	2B	(0.1–3.9)	0.02	4.7	0.15	-	-	-	0.03	6.1	0.19
*QGbg.mgb-2B.2*	IWB23783	2B	(29.9–47.9)	0.02	3.8	0.12	0.03	3.1	0.10	-	-	-
**Protein content**												
*QGpc.mgb-1B.1*	IWB41924	1B	(81.9–84.5)	0.64	3.0	0.10	-	-	-	-	-	-
*QGpc.mgb-2B.1*	IWA544	2B	(0.1–5.9)	-	-	-	−0.53	4.1	0.13	-	-	-
*QGpc.mgb-3B.1*	IWB66842	3B	(11.7–14.0)	-	-	-	0.22	4.8	0.15	-	-	-
*QGpc.mgb-3B.2*	IWB71842	3B	(126.0–130.9)	0.18	3.6	0.12	-	-	-	-	-	-
*QGpc.mgb-4A.1*	IWB71180	4A	(57.1–62.0)	−0.19	3.7	0.12	-	-	-	-	-	-
*QGpc.mgb-5A.1*	IWB28350	5A	(63.4–80.5)	−0.43	4.3	0.14	−0.38	5.4	0.17	-	-	-
*QGpc.mgb-7A.1*	IWB20381	7A	(1.9–15.9)	-	-	-	−0.31	5.6	0.17	-	-	-
*QGpc.mgb-7B.1*	IWB71499	7B	(53.9–61.9)	0.27	3.6	0.12	0.56	6.3	0.29	0.36	3.3	0.12
**Grain yield per spike**												
*QGys.mgb-1A.1*	IWB47651	1A	(8.3–16.0)	0.15	7.1	0.22	-	-	-	0.24	9.5	0.28
*QGys.mgb-2B.1*	IWA8152	2B	(19.9–37.9)	-	-	-	0.07	3.0	0.10	-	-	-
*QGys.mgb-3A.1*	IWB69601	3A	(5.5–12.5)	0.09	9.3	0.28	0.10	5.3	0.17	0.09	4.7	0.15
*QGys.mgb-3A.2*	IWB48828	3A	(42.0–50.0)	−0.06	4.5	0.14	−0.12	8.1	0.24	-	-	-
*QGys.mgb-3B.1*	IWB64877	3B	(20.6–28.5)	0.08	3.6	0.12	0.13	4.1	0.13	-	-	-
*QGys.mgb-3B.2*	IWB25495	3B	(72.0–84.0)	−0.06	4.1	0.13	-	-	-	-	-	-
*QGys.mgb-6B.1*	IWB23659	6B	(26.0–32.1)	−0.08	7.0	0.21	-	-	-	−0.09	5.5	0.17
**Heading time**												
*QHt.mgb-2A.1*	IWB54033	2A	(27.4–49.3)	−9.96	25.5	0.58	−10.48	26.0	0.59	−9.31	23.8	0.56

**Table 5:** Putative candidate genes associated with QTL for BG, GYS and HT detected in Duilio × Avonlea RIL population. Gene name, enzyme, contig, marker name and ID, type of mutation and localization on D × A consensus map and the durum consensus map (Maccaferri et al., [15]) are listed for each associated gene trait.

Trait	Gene	Enzyme	Contig	Marker ID	SNP	Wheat Map Position		
						Chrom	D × A Map	Durum Map
**β-glucan content**	*GLU1a*	β-glucosidase 1a	contig_2BL_8006334	IWB23783	T/C	2B	42.3	45.9
**Grain yield per spike**	*TaAP2*	APETALA2	contig_2BS_5227696	wmc213-2B	-	2B	-	31
			contig_2BS_5208541	wmc243-2B	-	2B	-	31.1
	*TaGI3*	gigantea 3	contitg_3AS_200_3324175	IWB65703	T/C	3A	50.6	51.5
	*Ta14A*	14-3-3 protein	contig_3B_10411870	IWB3714	A/G	3B	31.5	33.2
			contig_3B_10411871	IWB11529	A/G	3B	31.6	33.5
**Heading time**	*Ppd-A1*	Photoperiod sensitivity	contig_2AS_5262553	IWB54033	A/C	2A	47.8	46.2

## Supplementary Materials

Supplementary materials can be found at www.mdpi.com/1422-0067/18/6/1329/s1.
